# The Results of Two-stage Revision for Methicillin-resistant Periprosthetic Joint Infection (PJI) of the Hip

**DOI:** 10.5704/MOJ.2003.003

**Published:** 2020-03

**Authors:** A Santoso, TR Yoon, KS Park, IB Anwar, P Utomo, B Soetjahjo, T Sibarani

**Affiliations:** 1 Department of Orthopaedic and Traumatology, Universitas Sebelas Maret, Solo, Indonesia; 2 Department of Orthopaedic Surgery, Chonnam National University Bitgoeul Hospital, Jeonnam, Republic of Korea

**Keywords:** hip joint, periprosthetic infection, methicillin-resistant, two-stage revision

## Abstract

**Introduction:**

Periprosthetic joint infection (PJI) of the hip due to methicillin-resistant bacteria is difficult to treat and remain a challenge for arthroplasty surgeon.

**Material and Methods:**

Retrospective review was done to the patients who received two-stage revisions with an antibiotic loaded cement-spacer for PJI of the hip between January 2010 to May 2015. We found 65 patients (65 hips) with positive culture findings. Eight patients were lost to follow-up and excluded from the study. Among the rest of the 57 patients, methicillin-resistant infection (MR Group) was found in 28 cases. We also evaluate the 29 other cases that caused by the other pathogen as control group. We compared all of the relevant medical records and the treatment outcomes between the two groups.

**Results:**

The mean of follow-up period was 33.7 months in the methicillin-resistant group and 28.4 months in the control group (p = 0.27). The causal pathogens in the methicillin-resistant group were: Methicillin-resistant Staphylococcus aureus (MRSA) in 10 cases, Methicillin-resistant Staphylococcus epidermidis (MRSE) in 16 cases and Methicillin-resistant coagulase-negative Staphylococcus (MRCNS) in two cases. The reimplantation rate was 92.8% and 89.6% in the methicillin-resistant and control group, respectively (p= 0.66). The rates of recurrent infection after reimplantation were 23.1% (6/26) in the methicillin-resistant group and 7.6% (2/26) in the control group (p= 0.12). The overall infection control rate was 71.4% (20/28) and 89.6% (26/29) in the methicillin-resistant and control group, respectively (p = 0.08). Both groups showed comparable baseline data on mean age, BMI, gender distribution, preoperative ESR/CRP/WBC and comorbidities.

**Conclusions:**

Two-stage revision procedure resulted in low infection control rate and high infection recurrency rate for the treatment of methicillin-resistant periprosthetic joint infection (PJI) of the hip. Development of the treatment strategy is needed to improve the outcome of methicillin-resistant periprosthetic joint infection (PJI) of the hip.

## Introduction

Periprosthetic joint infection (PJI) is a difficult complication following total hip arthroplasty (THA). It is one of leading cause of morbidity and revision after total joint replacement surgery. Commonly the PJI of the hip cases were caused by antibiotic resistant-bacteria. Methicillin-resistant bacteria is one of most common cause^1^. The incidence of periprosthetic joint infection (PJI) of due to methicillin-resistant bacteria has been increasing^1, 2^. Previous study reported it could occur up to 50% of cases^3^. The treatment of periprosthetic joint infection associated with methicillin-resistant bacteria is even more challenging, as it is associated with high risk of prolonged and failure of treatment, patient morbodity and mortality^2, 4^. Furthermore, it is also associated with higher cost of treatment and resulted in a higher economic burden for orthopaedic community^1^.

Previous literatures showed that two-stage revision surgery remains the gold standard for surgical treatment of chronic PJIs, especially when the causative organism is a resistant-bacteria^3, 5^. We also prefer to perform two-stage revision THA for managing this case. Two-stage revision surgery is consisted of debridement and removal of the previous prosthesis followed by application of antibiotic-loaded cement spacer at the first stage. The use of articulated antibiotic-loaded cement spacer made the hip soft tissue maintained its tension. It also allows the patients to maintain the hip function during the interval between stages while waiting for the infection to completely healed before revision THA surgery as the second stage performed. In our current study, we tried to evaluate the outcome of two-stage revision surgery for PJI of the hip due to methicillin-resistant bacteria performed at our center.

## Materials and Methods

We retrospectively review the patient with hip PJI who treated between January 2010 and May 2015. We found 65 PJI hip cases (65 patients) in the period with positive culture result which were treated with the two-stage revision surgery. Eight patients were excluded due to loss to follow-up. Among the rest of the 57 patients, methicillin-resistant infection (MR Group) was found in 28 cases. For comparison, we also evaluate the 29 other cases that caused by the other pathogen (control group). The diagnosis of PJI was made based on the criteria from the workgroup of the Musculoskeletal Infection Society/MSIS^6^, where the patient who meets one major or more than three minor criteria was diagnosed as having PJI. The major criteria include: the presence of a sinus tract communicating with the prosthesis and growth of the microorganism from at least two separate tissue or joint fluid specimens from the affected prosthetic joint. The minor criteria include: elevated erythrocyte sedimentation rate (ESR), elevated C-reactive protein (CRP), elevated synovial white blood cell count (WBC), elevated synovial neutrophil percentage, presence of purulence in the affected joint, isolation of a microorganism in one culture of tissue or fluid, and more than five neutrophils per high-power field on histopathologic examination. The mean age of participating patients was 68.9 years (range, 49-82 years) in the methicillin-resistant and 65.9 years (range, 36-84 years) in the control group (*p=*0.37). The proportion of male and female gender between the two group was no different (15/13 vs 17/12, Male/Female in the methicillin-resistant and control group, respectively) (Table I). We compared all of the relevant medical records and the treatment outcomes between the two groups.

**Table I:** Baseline data of Methicillin-resistant group versus control group CRP: C-reactive protein, ESR: erythrocyte sedimentation rate; WBC: white blood count; THA: total hip arthroplasty; I&D: irrigation and debridement; COPD: chronic obstructuve pulmonary disease; HHS: Harris hip score.

Variable	Methicillin-resistant (Infection group n: 28)	Control (group n: 29)	p-value
Age (years)	68.9 (range, 49-82)	65.9 (range, 36-84)	0.37
Gender			0.70
Male	15	17	
Female	13	12	
Body mass index (kg/m2)	23.4 (range,19-34)	23.6 (range, 16-34)	0.81
CRP (mg/l)	5.2 (range,0.37-18.1)	5.9 (range, 0.5-24)	0.63
ESR (mm/hr)	68.3 (range 9-116)	70.5 (range, 11-118)	0.78
WBC (x103/ml)	11.6 (range,7.1-19.5)	13.0 (range,4.2-23.2)	0.24
Follow-up period (months)	33.7 (range, 12-67)	28.4 (range, 12-73)	0.27
Type of infected hip arthroplasty			0.01
Primary THA	3	18	
Revision THA	11	2	
Bipolar hemiarthroplasty	14	9	
Pre-operative antibiotic treatment			0.38
Yes	5	8	
No	23	21	
History of previous I&			0.97
Yes	2	2	
No	26	27	
Pre-operative HHS	50.1 (range, 21-71)	42.1 (range, 11-72)	0.03
Comorbidities			
Hypertension	14	9	
Diabetes mellitus	5	4	
Cerebrovascular disease	1	1	
Cardiovascular disease	2	3	
Renal insufficiency	5	3	
COPD	3	1	
Liver disease	2	2	
Malignancy	1	2	

**Fig. 1:**
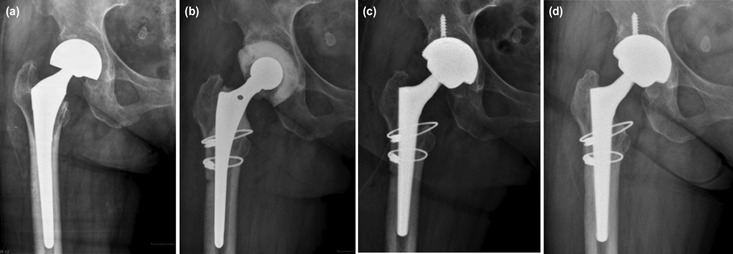
(a) Infected bipolar right hip hemiarthroplasty in a female 59-year-old due to MRSE. (b) Insertion of antibiotic cement spacer, extended trochanteric osteotomy was needed to remove the infected femoral stem. (c) Revision THA was performed after 54 days interval period. (d) Radiograph of final follow-up at 33 months, patient has excellent clinical outcome with no sign of infection.

In Surgical Technique, two-stage revision surgery consisted of drainage, removal of the prosthesis, debridement and implantation of an antibiotic loaded cement spacer on the first-stage surgery. A smaller, presterilised prostheses retrieved from previous cases were used to provide an endoskeleton for the spacers. A collar of an antibiotic-loaded cement (1 pack, 40g) was given to the femoral component, while the acetabular components were made by inserting a bolus of cement (1 pack, 40g) into the acetabular cavity and molded into the shape of the cup (Fig.1). The choice of the antibiotics depended on the results of the pre-operative bacterial culture. Two grams of Vancomycin [Hanomycin®, Samjim pharmaceutical, Seoul, Korea] and one gram of a Piperacilin-Tazobactam mixed antibiotic [Tazocin®, Wyeth Pharm, Seoul, Korea] were used on each acetabular and femoral cement spacer if pre-operative culture were methicillin-resistant bacteria. Antibiotic Simplex cement [Stryker, Allendale, NJ] impregnated with erythromycin was used for both the femoral and the acetabular component. A negative suction drain was used after wound closure. Postoperative intravenous antibiotic treatment was given at minimum 2 weeks and followed by oral antibiotic for 2-4 weeks. However, this can be varied based on the laboratory results. Post-operative antibiotics were selected in consultation with the Department of Infectious Disease. Vancomycin alone or in combination with other antibiotic were used in all methicillin-resistant group post-operatively. While cephalosporin (either first or third generation) were the most commonly (62%) used antibiotic in control group. Total WBC counts, ESR, and CRP levels were measured at weekly intervals for the first month and then at four weeks intervals post-operatively. Healing of the wound and sinus (if any), return of CRP and ESR levels to normal and/or medical fitness for surgery were the criteria to proceed to the second stage revision THA^7^.

At the second stage revision THA surgery, a cementless prosthesis was preferred on both the femoral and acetabular components. However, cemented fixation was also done for acetabular or femoral reconstruction whenever it was not possible to perform cementless fixation. Post-operatively, first-generation cephalosporin antibiotics were administered for three to five days. Patients were allowed to walk with a walker or crutches as soon as their conditions permitted. Radiographs were obtained, and total WBC, ESR, and CRP levels were repeatedly measured at 1, 3, 6, and 12 months post-operative follow-up visit. Harris hip scores was used to evaluate clinical outcomes of the surgery^8^. The state of controlled or recurrent infection was also following the previously used MSIS diagnostic criteria. The SPSS software, version 17.0 (SPSS Inc., Chicago, IL, USA) was used to evaluate all data. The Chi-square test was used to compare all of the categorical data, while the Student’s t-test was used to compare numerical data between the methicillin-resistant and control group. Statistical significance was defined when p values <0.05.

## Results

There were no differences on baseline data between the two groups on age, gender, BMI, pre-operative laboratory (CRP, ESR,WBC), pre-operative antibiotic treatment, history of irrigation and debridement and follow-up period between the group (P>0.05). However, a significant difference was found on type of infected hip prosthesis, which the infected revision total hip arthroplasty and infected bipolar hemiarthroplasty were found predominantly on the MR group, while the infected primary total hip was predominantly found on the control group (p<0.05) (Table I).

The mean of follow-up was 33.7 months in the MR group and 28.4 months in the control group (p>0.05). The reimplantation rate after antibiotic cement spacer was 92.8% and 89.6 % in the MR group compared to control group, respectively. Most of the patient received single time cement spacer (88.5%;23/26 VS 92.3%; 24/26, respectively). Two patients in the MR group and one patient in the control group received >2 times of spacer to obtain infection control. Among patients who received reimplantation, recurrency of infection occurred in 23.1% in the MR group, while the control group was 7.6% (p>0.05). Therefore the overall infection control rate at the final follow-up was 71.4% (20/28) in the MR group and 89.6% in the control group (26/29) (p> 0.05). The interval period between the first and the second stage of surgery was mean 151 vs 144 days in the MR and control group, respectively (p>0.05). While the time needed to normal CRP was mean 112 vs 105 days in the MR and control group, respectively (p>0.05). Despite there was a difference (p<0.05) on pre-operative functional based on Harris hip score between the two groups at the pre-operative state, no difference was found on post-operative functional evaluation between the two groups at the final follow-up (p>0.05). The complication rate at the spacer period and after reimplantation also comparable between the group (p>0.05) (Table II).

**Table II:** Treatment results of two-stage revision of Methicillin-resistant group versus control group

Treatment Results	Methicillin-resistant group (n:28)	Control group (n:29)	p-value
Interval period between stage (days)	151.4 (range, 33-616)	144.2 (range, 28-584)	0.84
Time to normal CRP (days)	112.8 (range, 11-601)	105 (range, 12-575)	0.83
Received reimplantation	26 (92.8%	26 (89.6%)	0.66
Single time cement spacer	23	24	
≥times cement spacer	2	1	
Spacer and Girdlestone	1	1	
Never reimplantation	2 (7.2%)	3 (10.4%)	
Spacer retention	1	3	
Convert to Girdlestone	1	0	
Recurrency of infection			
(after reimplantation)	6 (23.1%)	2 (7.6%)	0.12
I&	0	0	
One-stage revision	1	0	
Two-stage revision	4	2	
Girdlestone	1	0	
Overall infection control rate	20/28 (71.4%)	26/29 (89.6%)	0.08
HHS at final follow-up	82.2 (range, 47-94)	83.6 (range, 67-95)	0.58
ETO during 1st stage			0.90
Yes	15	16	
No	13	13	
Adverse events during spacer period			>0.05
Spacer dislocation	2	4	
Periprosthetic fracture	5	6	
Cement dislodgement	2	4	
Adverse events after reimplantation			>0.05
Aseptic loosening	1	0	
Dislocation	2	2	
Periprosthetic fracture	0	1	

CRP: C-reactive protein, I&D: irrigation and debridement; HHS: Harris hip score; ETO: extended trochanteric osteotomy.

Methicillin-resistant staphylococcus epidermidis (MRSE) was the most common bacteria founded in the MR group (16/28, 57.1%), followed by Methicillin-resistant staphylococcus aureus (MRSA) by 35.7% (10/28) (Table III). In the control group, infection due to Methicillin-sensitive staphylococcus aureus (MSSA) and multiorganism infection were the most common occur (both 20.6%, 6/29 and 20.6%, 6/29). Three cases of MRSE and three cases of MRSA experienced recurrency of the infection. While in the control group, recurrency occurred due to Streptococcus sanguinis and a multiorganism infection.

**Table III:** List of isolated pathogen in Methicillin-resistant group versus control group

Isolated pathogen	MR infection	Other infection	Number of reimplantation	Number of recurrency
MRSE	16	-	16	3
MRSA	10	-	8	3
MRCNS	2	-	2	-
MSSA	-	6	6	-
Staphylococcus capitis	-	3	3	-
Streptococcus haemolyticus	-	1	1	-
Proteus mirabilis	-	1	1	-
Pseudomonas aeruginosa	-	5	4	-
Corinobacterium sp.	-	1	1	-
Escherecia coli	-	1	1	-
Streptococcus Sanguinis	-	1	1	1
Acinetobacter Baumani	-	1	1	-
Klebsiella Pneumonia	-	1	1	-
Candida Albicans	-	1	-	-
Mycobacterium sp.	-	1	-	-
Multiorganism	-	6	6	1
MRSA: Methcillin-resistant Staphylococcus	aureus; MRSE:	Methicillin-resistant Staphyl	ococcus epidermidis;	MRCNS: Methicillin-

MRSA: Methcillin-resistant Staphylococcus aureus; MRSE: Methicillin-resistant Staphylococcus epidermidis; MRCNS: Methicillin-resistant coagulase-negative Staphylococcus; MSSA: Methicillin-sensitive Staphylococcus aureus

## Discussion

As the advancement of medicine and surgery, it is known that methicillin-resistant bacteria has been becoming one of the major causes of various medical devices related infection^9^. Several orthopaedic centers have performed a preoperative screening protocol for the carriers of methicillin-resistant bacteria^10, 11^. Unfortunately, this procedure remains resulted high rates of missed carriers. It has been reported with the standard swab and culturing procedure still couldn’t identify up to one-third of patient with carriage of MRSA/MSSA^10^. Furthermore, the pre-operative decolonisation protocol prior to elective total joint arthroplasty also still failed to decolonise the MRSA until 22% of the cases and resulted no difference on infection risk between the protocolised and the control group^11^. This could make the rate of methicillin-resistant related infection remain high.

Several previous studies reported the result of two-stage revisions for the treatment of chronic hips/knee PJI. Leung *et al* reported the infection control rate of MRSA/MRSE related PJI of the hip which treated with two-stage revision was 79%^5^. Almost similarly, Parvizi *et al* reported two-stage revision could control the infection of methicilllin-resistant hip PJI by 75% and knee PJI by 60%, whereas debridement can only control the infection by 37%^12^. Our recent study even resulted a lower infection control rate in the methicillin-resistant group by 71%. We also found the infection recurrency rate after the second stage of procedure was 23%, this was higher compared to the previous studies reported by Ryu DJ *et al*^13^ which recurrency rate after hip/knee PJI treated with two-stage revision was 13.9% and Leung *et al*^5^ which recurrency of hip PJI by 21%. All (six cases) of our recurrent of infection cases in methicillin-resistant group has similar bacterial cause of infection with the prior status (three MRSA, three MRSE). This finding also similar with the study by Parvizi *et al* which found similar bacterial cause of infection in all (eight cases) their recurrent cases^12^. This could indicate that the methicillin-resistant infection eradication might be inadequate at the first stage of surgery and/or possibly the decision to perform second-stage surgery was inaccurate. The criteria for decision to perform the second stage of surgery sometimes varied. Whether reimplantation has to be performed or delayed when the infection marker start to normal remains on debate among arthroplasty surgeons. We found our interval between stages of the surgery was mean 151 days (±5 month) which was not significant difference to the control group (144 days/±4.8 month). This was shorter than the study reported by Leung *et al*^5^ with mean interval period of six months (range, 2-15 months). Most of the surgeons use CRP, ESR and WBC as the marker of eradication of infection prior to perform the second stage of surgery. We may combine with the use of procalcitonin and/or α-defensin as the marker of eradication of the infection^14^. A rapid polymerase-chain reaction (PCR) assay (ie Xpert® MRSA/SA ) also might be beneficial for assessment of intra-operative methicillin-resistant infection status^15, 16^. All of those assays were not done in our centre, thus an overestimation of the eradication status may be occurred. Application of a strict criteria for reimplantation in two-stage revision for MR-PJI treatment is needed to improve the infection control rate.

The resistence of bacteria to the common antibiotic also might be one of the reasons in the difficulty to eradicate methicillin-resistant infection. Vancomycin is the most commonly used antibiotic for the treatment of methicillin-resistant PJI, it was also true in our recent study. Vancomycin can be used as a local antibiotic embedded in cement-spacer or a systemic treatment. Combined antibiotic treatment or discovering the new antibiotic for the methicillin-resistant bacteria might be necessary to increase the success rate of the treatment.

This study has several limitations include the retrospective analysis with small number of cases which has its own weakness. Although this study involved only relatively short follow-up period, still we believe the results of this study could estimate the benefit of two-stage revision for the treatment of methicillin-resistant PJI of the hip.

## Conclusions

Two-stage revision procedure resulted low infection control rate and high infection recurrency rate for the treatment of methicillin-resistant periprosthetic joint infection (PJI) of the hip. Development of the treatment strategy is needed to improve the outcome of methicillin-resistant periprosthetic joint infection (PJI) of the hip.
